# Atherosclerosis in Rheumatoid Arthritis

**DOI:** 10.1155/2012/489608

**Published:** 2012-10-17

**Authors:** Miguel A. González-Gay, Zoltan Szekanecz, Calin D. Popa, Patrick Dessein

**Affiliations:** ^1^Division of Rheumatology, Hospital Universitario Marqués de Valdecilla, IFIMAV, 39008 Santander, Spain; ^2^Department of Rheumatology, Institute of Medicine, University of Debrecen Medical and Health Sciences Center, 98 Nagyerdei Street, Debrecen H-4032, Hungary; ^3^Department of Rheumatology, Radboud University Nijmegen Medical Centre, P.O. Box 9101, 6500 HB Nijmegen, The Netherlands; ^4^Research Unit of Cardiovascular Pathophysiology and Genomics, School of Physiology, Faculty of Health Sciences, University of the Witwatersrand and Charlotte Maxeke Johannesburg Academic Hospital, Johannesburg 2193, South Africa

There is a growing body of evidence supporting an increased risk of cardiovascular (CV) mortality in patients with rheumatoid arthritis (RA). This is the result of complex mechanisms leading to accelerated atherosclerosis [1]. In this regard, besides classic CV risk factors [2], chronic inflammation [3] and a genetic component [3, 4] have been proposed to influence the development of atherosclerosis in RA.

This special issue encompasses different aspects of the CV disease associated to RA. With respect to this, the link between atherosclerosis and RA was discussed. Surrogate markers of atherosclerosis have been found to be useful in predicting the presence of atherosclerosis disease in subclinical stages in adults with RA [5, 6]. Data shown in this special issue also confirmed that children with juvenile idiopathic arthritis may have higher carotid intima-media wall thickness index values than controls. These observations emphasize the need for increased awareness of the risk of atherosclerosis in children with juvenile idiopathic arthritis.

An exhaustive literature review on the genetic influence in the development of CV disease in patients with RA was also included. With respect to this, it is important to keep in mind that besides gene polymorphisms located within the MHC region [3, 4], variations of genes located outside this region, such as *CCR5*, *MTHF* [7, 8], are of potential relevance in the increased risk of CV disease associated to RA.

Adipokines are molecules not only implicated in the development of metabolic syndrome but also in inflammatory mechanisms that may play a role in the pathogenesis of different autoimmune diseases [9, 10]. A timely review on the implication of adipokines in the development of the atherosclerotic disease not only focusing on RA but also discussing the link between these molecules and the presence of atherosclerosis in other chronic inflammatory rheumatic diseases was included in the special issue.

The mechanisms associated with endothelial dysfunction, an early step in the atherogenesis process, in patients with RA are far from being completely understood. In this special issue an assessment of the potential role of asymmetric dimethylarginine and apelin as biomarkers to detect early data of endothelial dysfunction in patients with RA was included.

Anti-TNF-*α* drugs constitute the mainstay of therapy in RA patients with severe disease that is refractory to conventional disease modifying antirheumatic drugs. One of the articles included in the special issue confirmed the previously reported short-term beneficial effect of the fully human-anti-TNF-*α* monoclonal antibody adalimumab on endothelial function [11]. In addition, new data showing persistent improvement of endothelial function in adalimumab-treated RA patients without progression of the carotid intima-media thickness are reported. This is of potential relevance as the use of anti-TNF-*α* therapy has been associated with a decrease of mortality in RA patients, mainly due to a reduction in the incidence of CV events [12].

In line with the above, Popa et al. had previously assessed the effects of the anti-TNF-*α* therapy on the HDL antiatherogenic function. They observed that infliximab, a chimeric anti-TNF-*α* monoclonal IgG1 antibody was able to improve HDL antioxidative capacity. This effect was sustained 6 months after anti-TNF-*α* therapy had been initiated [13]. In this special issue the same group conducted an extensive review on the effect that different biologic therapies exert on the atherogenic index and HDL cholesterol in patients with RA.

Taken together, these studies confirm the presence of complex mechanisms that influence the development of atherosclerosis in RA. Nevertheless, further studies are needed to shed light on the problem of augmented risk of CV disease in patients with RA.

*Miguel A. González-Gay* *Miguel A. González-Gay* *Zoltan Szekanecz* *Zoltan Szekanecz* *Calin D. Popa* *Calin D. Popa* *Patrick Dessein* *Patrick Dessein*
